# Stimulators of immunogenic cell death for cancer therapy: focusing on natural compounds

**DOI:** 10.1186/s12935-023-03058-7

**Published:** 2023-09-13

**Authors:** Mina Amiri, Ommoleila Molavi, Shahnaz Sabetkam, Sevda Jafari, Soheila Montazersaheb

**Affiliations:** 1 Department of Pharmaceutical Biotechnology, Faculty of Pharmacy, Tabriz University of Medical Sciences, Tabriz, Iran; 2 Department of Anatomy, Faculty of Medicine, university of Kyrenia, Kyrenia, Northern Cyprus; 3 Department of Anatomy and histopathology, Faculty of medicine, Tabriz medical sciences, Islamic Azad University, Tabriz, Iran; 4 Nutrition Research Center, Tabriz University of Medical Sciences, Tabriz, Iran; 5 Molecular Medicine Research Center, Tabriz University of Medical Sciences, Tabriz, Iran

**Keywords:** Immunogenic cell death (ICD), DAMPs, Natural compounds, Synthetic agents

## Abstract

A growing body of evidence indicates that the anticancer effect of the immune system can be activated by the immunogenic modulation of dying cancer cells. Cancer cell death, as a result of the activation of an immunomodulatory response, is called immunogenic cell death (ICD). This regulated cell death occurs because of increased immunogenicity of cancer cells undergoing ICD. ICD plays a crucial role in stimulating immune system activity in cancer therapy. ICD can therefore be an innovative route to improve anticancer immune responses associated with releasing damage-associated molecular patterns (DAMPs). Several conventional and chemotherapeutics, as well as preclinically investigated compounds from natural sources, possess immunostimulatory properties by ICD induction. Natural compounds have gained much interest in cancer therapy owing to their low toxicity, low cost, and inhibiting cancer cells by interfering with different mechanisms, which are critical in cancer progression. Therefore, identifying natural compounds with ICD-inducing potency presents agents with promising potential in cancer immunotherapy. Naturally derived compounds are believed to act as immunoadjuvants because they elicit cancer stress responses and DAMPs. Acute exposure to DAMP molecules can activate antigen-presenting cells (APCs), such as dendritic cells (DCs), which leads to downstream events by cytotoxic T lymphocytes (CTLs) and natural killer cells (NKs). Natural compounds as inducers of ICD may be an interesting approach to ICD induction; however, parameters that determine whether a compound can be used as an ICD inducer should be elucidated. Here, we aimed to discuss the impact of multiple ICD inducers, mainly focusing on natural agents, including plant-derived, marine molecules, and bacterial-based compounds, on the release of DAMP molecules and the activation of the corresponding signaling cascades triggering immune responses. In addition, the potential of synthetic agents for triggering ICD is also discussed.

## Introduction

**Fig. 1:**
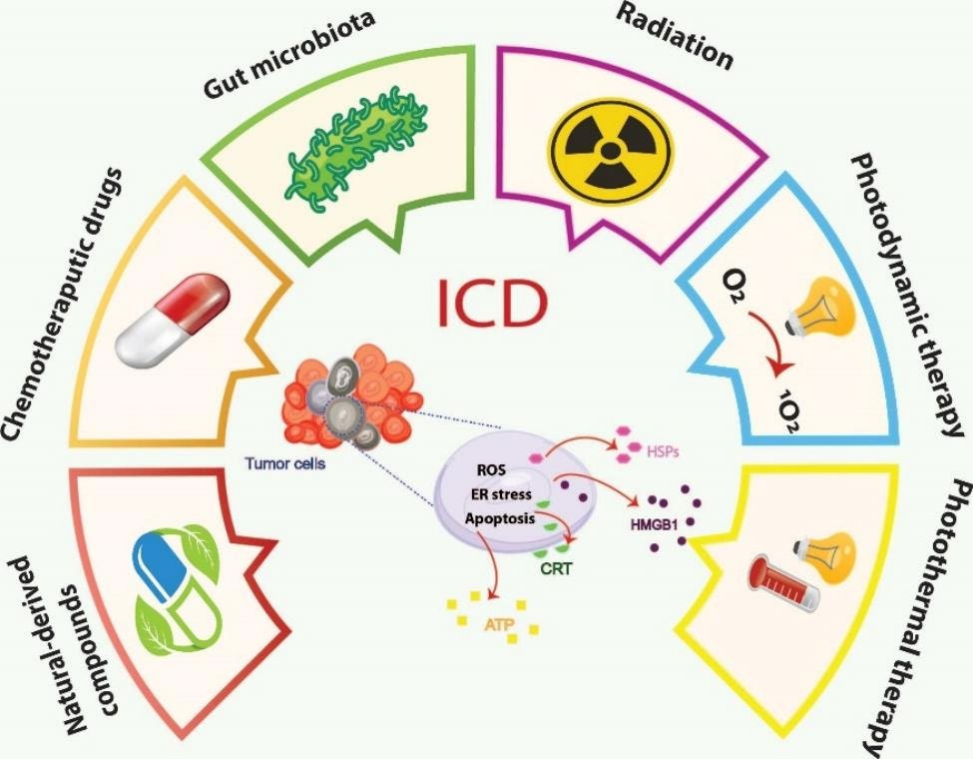
Immunogenic cell death (ICD) inducers can be categorized into different groups, including natural-derived compounds, chemotherapeutic agents, gut microbia, radiotherapy, photodynamic therapy and photothermal therapy

A condition known as cancer is among the most widespread death reasons in the world today. Based on the reported data, the incidence of cancer in 2020 was more than 19.3 million cancer cases word wide that led to approximately 10 million deaths [1]. Cancer is characterized by uncontrollable and excessive cell divisions [2]. Growing evidence has shown that cancer development is considerably affected by immune deficiency. Cancer recurrence and metastasis can be prevented by stimulating and mobilizing the immune system. This process, called immunogenic cell death (ICD), occurs when factors that promote immunity activation are released. In ICD, dying cancer cells release pathogen-associated molecular patterns (PAMPs) and danger-associated molecular patterns (DAMPs). Recruitment and activation of dendritic cells (DCs) occur after recognition by pattern recognition receptors (PRRs), which recruit DCs to eradicate cancer cells through phagocytosis. T-cells are activated by DCs and stimulating signals. Therefore, T-cells and DCs play a crucial role in ICD [3]. ICD must be accompanied by a series of immunomodulatory events, including translocation of calreticulin (CRT), the release of high mobility group box-1 (HMGB-1), adenosine triphosphate (ATP), heat shock proteins (HSPs), and ANXA1. These mediators can act by enhancing the immune responses against tumor cells [4]. It is well-known that radiotherapy, chemotherapy, and photodynamic therapy can activate ICD in tumor cells [5]. Amongst the different types of ICD inducers (Fig. 1), natural compounds have gained much attention due to their low toxicity and potent anticancer impacts that are mediated by inhibiting various pathways involved in cancer development. Several reports have shown that natural compounds can alter the immunosuppressive tumor microenvironment (TME) to immunogenic ones and induce ICD when combined with chemotherapy drugs or alone. In other words, natural agents may have the potential to increase efficacy of cancer immunotherapy [6, 7]. This review explores various ICD-inducing natural compounds to show their efficacy in anticancer responses to prevent cancer metastases and recurrences.

## Immunogenic cell death: an innovative approach to cancer therapy

Globally, cancer remains one of the most significant barriers to extending life expectancy. As estimated by the World Health Organization (WHO) in 2019, cancer has become one of the biggest causes of death among those aged 70 and younger [8, 9]. The immune system plays a crucial role in regulating tumor progression by detecting and eradicating tumor cells. Notably, immune cells are trained to not respond to normal cells. In contrast, tumor cells sometimes exhibit mutations that produce tumor-specific antigens, recognized by the immune system as a foreign agent that leads to the death of cancer cells. “Antitumor immunity” relates to adaptive and innate responses that regulate the growth of tumors Antitumor effects result from the interaction of various innate and adaptive immune factors like T and B cells, APCs, and NKs [10]. Despite the antitumor activity of immune cells, tumor-induced immunosuppression is a major obstacle in this regard. Tumors can disrupt the balance between the compartments of the regulatory and effector cells to escape immune recognition and subsequent eradication [11, 12]. In recent years, many anticancer therapies have been developed to induce ICD that alert the immune system to dying cancer cells [13, 14]. T cell-mediated immunity is triggered by ICD, a unique type of cell death that responds to antigens produced by dead cells. Several mechanisms contribute to the stimulation of ICD through the translocation of or release of DAMPs by dying cells to trigger an immune response [14]. Many studies have shown that ICD is an interesting approach to activating anticancer immunity. Upon exposure to a variety of specific stressors, cancer cells undergoing ICD and dying cells release DAMPs that produce neoantigens and stimulate adaptive immunity. In this context, ICDs can mediate vaccine-like traits in cancer. As a result, the inducement of ICD can mimic cancer vaccination [15]. This mechanism is mediated by enhancing the immune response against cancer by maturing DCs, activating CTLs, and increasing NK cell function [16]. DAMPs are present in living cells and show immunostimulatory activities in dying cells. As a result, a wide range of DAMPs is being investigated as potent diagnostic or therapeutic agents in cancer therapy [14].

### ICDs’ key hallmarks

**Fig. 2:**
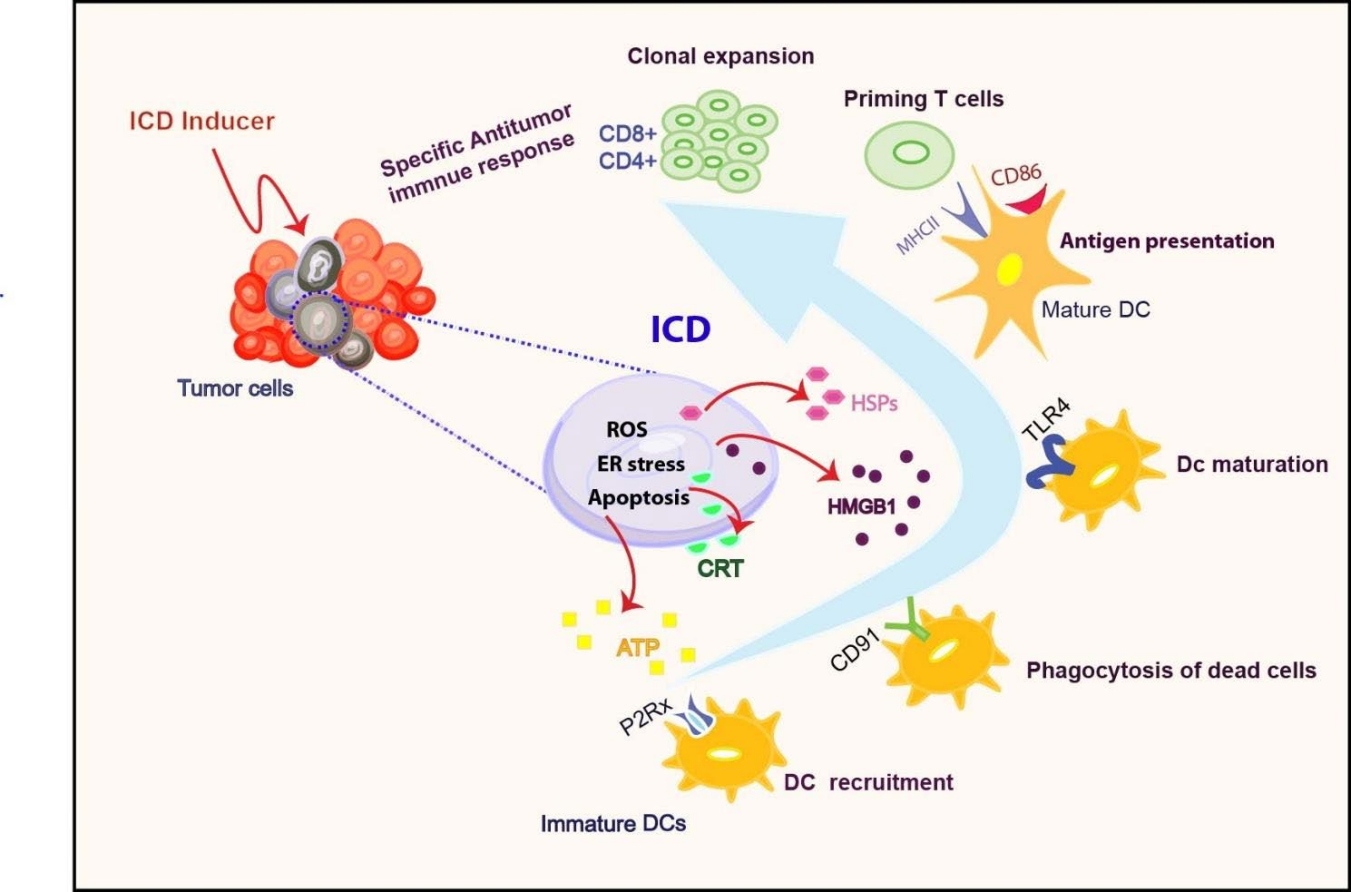
Immunogenic cell death (ICD) inducer causes regulated kind of cell death through ROS production and endoplasmic reticulum (ER) stress in tumor cells. Dead tumor cells expose or release damage associated molecular patterns (DAMPs) such as adenosine Triphosphate (ATP), calreticulin (CRT), High mobility group box protein 1(HMGB1) and Heat shock proteins (HSPs). DAMPs cause functional maturation of dendritic cells through binding to their specific receptors. Mature DCs present cancer specific antigens to T cells and activate anticancer immune response against remained cancer cells

As discussed earlier, the ICD process involves the release of intracellular molecules, DAMPs. DAMPs have immunostimulatory properties when exposed to or secreted by dying cells. DAMPs are excreted during necrosis under inflammatory or pathological conditions. Several lines of evidence show that cancer cells exposed to chemotherapy or radiotherapy can produce DAMPs, recruit, and activate immune cells. Additionally, DAMPs stimulate phagocytosis and serve as triggers for CTLs to kill cancer cells [3, 17, 18]. In ICD, DAMPs are produced under endoplasmic reticulum (ER) stress and triggered by reactive oxygen species (ROS) [19]. Based on their localization or release site, three significant subclasses of DAMPs exist [1] cell surface-appearing DAMPs (e.g., HSP 90, HSP70, CRT), [2] extracellular appearing DAMPs (e.g., proinflammatory cytokines (like tumor necrosis factor-α (TNF-α), interleukin 10 (IL-10), IL-8, HMGB1, and uric acid), and [3] end-stage degradation products (e.g., ATP, DNA, and RNA) [20, 21]. Several receptors contribute to the recognition of DAMPs, including the toll-like receptor (TLR) family, retinoic acid-inducible gene-I (RIG-I) receptors, and NOD-like receptors (NLRs) [22, 23] (Fig. 2). Over the past decade, several clinical trials have been initiated to determine the efficacy of DAMPs in cancer treatment. Clinical studies have shown that patients with higher HSP and CRT exposure in response to ICD inducers are more likely to survive [24]. Higher levels of HMGB1 in patients with esophageal squamous cell carcinoma were associated with increased survival in patients receiving chemoradiotherapy [25].

#### Calreticulin (CRT)

CRT is the most well-known DAMPs found in the ER lumen. CRT plays various roles in immunity, such as maintaining Ca2 + homeostasis and chaperone activity. CRT also regulates cell proliferation, protein synthesis, cell invasion, cell adhesion, and nuclear transport. Gardai et al. showed that CRT acts as a general recognition ligand on the surface of apoptotic cells [26]. There is increasing evidence that CRT contributes significantly to antitumor immunity and the immunogenicity of dying cells [27]. It was found that the induction of ICD causes ER stress and leads to the release or exposure of immune cells to DAMP molecules such as CRT. Upon ICD induction, CRT exposure requires downstream ER stress-responsive genes, including caspase-8-mediated cleavage of ER-resident protein 57 (ERp57), stimulation of BCL2-associated X protein (Bax), Bcl-2 homologous antagonist/killer (Bak), and B cell receptor-associated protein 31 (BAP31). ERp57 mediates the transport of CRT to the plasma membrane; thereby, the deletion of ERp57 prevents CRT translocation [27–29]. ICD inducers are divided into two types: Type I, which elicits danger signaling through non-lethal ER stress such as chemotherapy, and Type II, which triggers danger signaling by ER stress such as hypericin-photodynamic therapy (Hyp-PDT) [30]. In the case of type II ICD inducers, fewer demands are needed because of the reliance of this type of inducer only on Bax, Bak, protein kinase-like ER kinase (PERK), and secretory pathways [28]. Following ER stress, CRT translocates to the outer surface of the cell and binds to specific receptors (including CD91) on the DCs surface, causing phagocytosis of dying tumor cells. To act as an “eat me signal,” CRT activates APCs [21]. According to Obeid et al., CRT induces apoptosis via anthracycline and mitoxantrone, and a positive correlation was observed between CRT exposure and inducer-mediated immunogenicity [31].

Moreover, mRNA expression of CRT in cancer cells affects the composition and density of infiltrating immune cells. Indeed, CRT expression is mainly linked to CTLs and DCs infiltration in various types of cancer, such as colorectal, ovarian, and breast cancers [32]. CRT is also targeted by miR-27a in colorectal cancer, as recently discovered. MiR-27a is a negative mediator of drug-induced ICD. This was mediated by reducing CRT levels and exposure to the cell surface. Exposure to CRT enhances the detection of tumor cells by CTLs and DCs [33, 34].

#### Heat-shock proteins (HSPs)

Another ICD hallmark is the heat-shock protein (HSP), which contributes to protein folding and refolding in stress states [35]. Upon exposure to stress (e.g., oxidative stress, irradiation, or chemotherapy agents), HSPs are overexpressed in the intracellular environment and transported to the plasma membrane [36]. There are many HSPs, but HSP70 and HSP90 are the most associated with ICD. HSP70 and HSP90 have different functions in cancer, depending on their location. Intracellularly located HSPs have defensive properties, whereas extracellular or membrane-bound HSPs have immunological functions [37]. Mobilizing intracellular HSP to the plasma membrane results in potent immunostimulant activity [38]. HSP70 and HSP90 display immunostimulatory activities under exposure to the extracellular layer of dying cells Ecto-HSP70 and HSP90 can interact with receptors on the surface APCs (e.g., CD40, CD91, and LOX1) and control the immunogenicity of dying cells. This leads to the cross-presentation of cancer cell antigens to MHC class I molecules and subsequent activation of CD8 + T-cells. Collectively, the surface exposure of HSPs to cancer cells facilitates anticancer immune responses by enhancing the immunogenicity of cancer cells [39].

#### High mobility group box 1 release (HMGB1)

Accumulating evidence has shown that extracellular HMGB1 can elicit anticancer immune responses during ICD. The HMGBs family is composed of three members: HMGB1, HMGB2, and HMGB3, all of which have the potential to bind and distort DNA molecules [40]. HMGB1 is a non-histone chromatin-binding protein, the first family member to be identified. The function of HMGB1 depends on its subcellular localization. The nucleus HMGB1 mediates various functions, including DNA repair, nucleosome maintenance, transcription, and recombination, whereas extracellular HMGB1 participates in angiogenesis and chemotaxis [41, 42]. Upon binding to the corresponding receptors located extracellularly, HMGB1 produces endogenous danger signals in adjacent cells and induces immunity and inflammation. HMGB1 is broadly expressed and acts as an extracellular signal upon active secretion by immune-associated cells or upon passive secretion by injured, dying, and dead cells. Indeed, HMGB1 has intracellular activities and contributes to multiple extracellular functions that are mediated by corresponding receptors, including TLR2, TLR4, and RAGE (receptors for advanced glycation end-products). HSPs and HMGB1 can induce a Th1 type of immune response against cancer cells through binding to TLR4 receptors on DCs [43]. Following its release during the death process, HMGB1 acts as an anti-inflammatory and immune-regulating DAMP. Anticancer agents, apoptosis, and ICD stimulation factors also influence the release of HMGB1. In immature DCs, extracellular HMGB1 binds to TLR4, causing DC maturation and CTL activation [44].

#### Adenosine triphosphate (ATP)

ATP is another DAMP released during ICD. ATP secretion from dying cells is a crucial factor for the effective initiation of ICD. In response to ICD inducers, ATP is redistributed from lysosomes to autolysosomes and is released by a mechanism that requires lysosomal-associated membrane protein 1 (LAMP 1), which ultimately translocates to the plasma membrane in a caspase-dependent manner. In addition, ATP secretion is mediated by caspases and pannexin 1 (PANX1). ICD requires PANX1 to translocate LAMP1 to the cell surface and exocytose lysosomes [45]. Extracellular ATP acts as a powerful chemotactic agent for APCs and their precursors by binding to purinergic receptors P2RY2 and P2RX7. Therefore, dying cells lose their immunogenic properties not only when ATP does not assemble in their microenvironment but also when P2RY2 or P2RX7 is not present in the myeloid compartment. P2RX7 signaling also activates the NLR domain-containing protein 3 (NLRP3) inflammasome, which in turn elicits the release of IL-1β, a cytokine involved in the extension of an antitumor immune response. Studies have shown that ATP acts as a chemoattractant for DC precursors. La Sala et al. reported that incubation of immature DCs with ATP (250 µM) for 24 h enhanced the level of CD54, CD80, CD83, and CD86 [46]. In the process of cell death, the apoptotic phase that stimulates ICD is necessary for ATP secretion [47].

#### Type I IFN

Recent studies have indicated that IFN type I is a crucial component of ICD. It plays a substantial role in innate and adaptive immunities [48]. In addition to stimulating antigen presentation, type I IFN enhances the antitumor activity of T cells [49]. Type I IFN response pathway genes are upregulated in tumor cells by factors that promote ICD. These include anthracyclines, oncolytic viruses, and radiotherapy. In addition, a type I IFN-related profile can predict the clinical responses to anthracycline-based chemotherapeutics. The chemokine ligand 10 (CXCL10) is an IFN-I-induced gene that acts as a chemotactic factor and recruits immune cells to eradicate cancer cells selectively [50, 51].

#### Annexin A1 (ANXA1)

ANXA1 binds phospholipids in the presence of calcium [52]. The immunosuppressive properties of ANXA1 can enhance DC function during ICD implantation. As previously mentioned, APCs interact with dying cancer cells, and ANXA1 is released from dying cancer cells. It binds to the formyl peptide receptor 1 (FPR1) on APCs [53, 54]. In other words, ANXA1 mediates the uptake of tumor antigens for presentation [55]. Neither anthracycline nor oxaliplatin showed therapeutic effects in tumor cells lacking ANXA1 or immune cells lacking FPR1 [56].

## ICD inducers: focusing on natural compounds

The first classification system for ICD inducers was proposed by Agostinis et al. in 2013. ICD inducers are classified into types I and II, according to their direct or indirect targeting of the ER [57]. As briefly described in previous sections, Type I ICD inducers (e.g., anthracyclines) do not function by targeting the ER directly but rather by targeting a variety of cellular compartments. In contrast, the ER is the primary target of Type II ICD inducers and directly enhances the immunogenicity of ICD. Only a few Type II inducers are available; for example, hypericin resides mainly in the ER and, when irradiated, generates ROS-driven ER stress, leading to ICD. Type II inducers stimulate ICD and result in a more effective immune response. Type II inducers usually generate higher levels of DAMPs. Most of the discovered ICD-induced compounds belong to Type I [58].

### Natural compounds

**Fig. 3:**
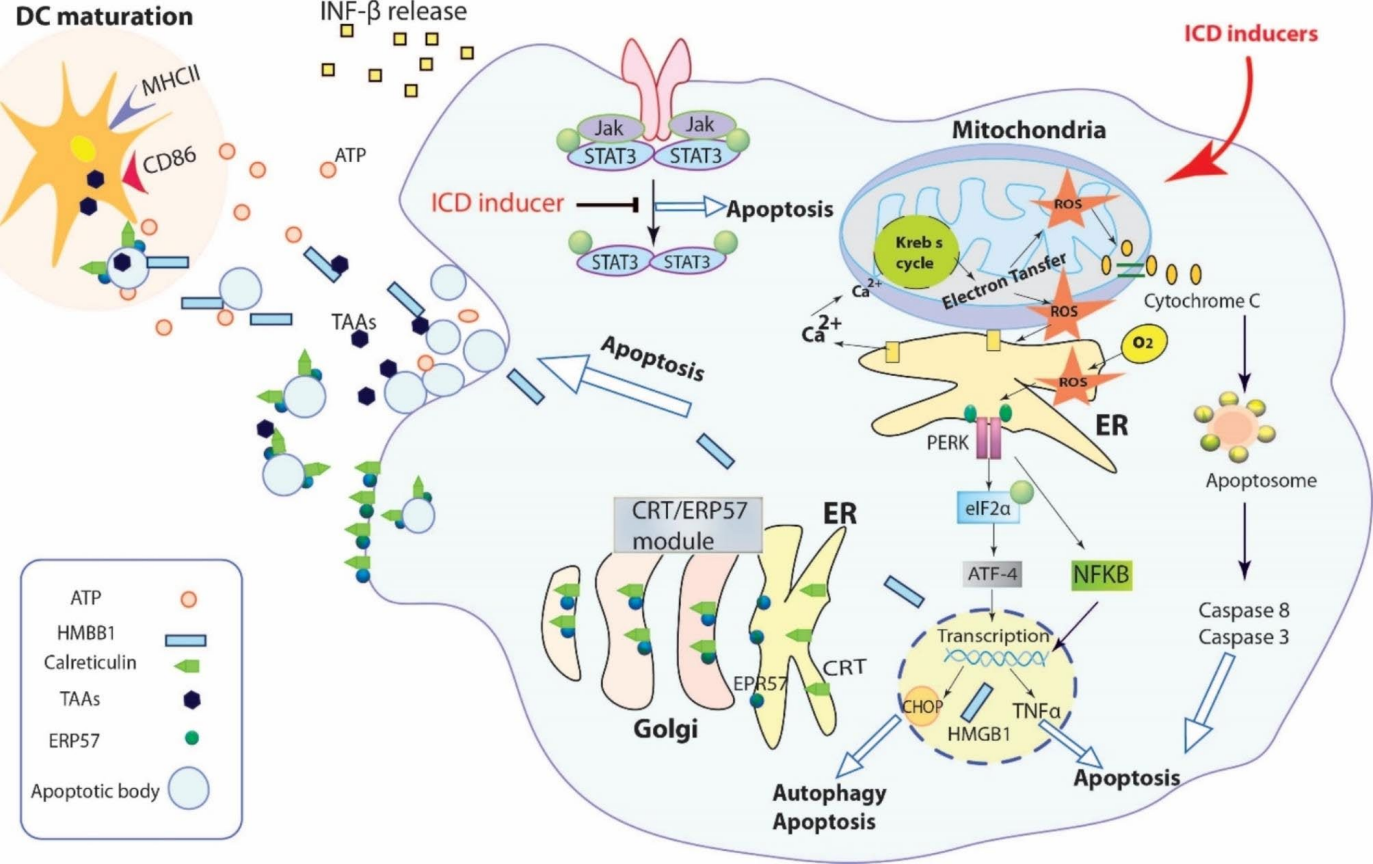
Natural compounds induce ICD through different mechanism leading to apoptotic type of cell death. Induction of oxidative stress in mitochondria and endoplasmic reticulum (RT) is the most common phenomena in ICD induced by natural agents. Releasing cytochrome C, activation of PERK/eIF2α/ATF4/CHOP pathway, prevention of NF-κB, increasing TNFα expression and inhibiting STAT3 activation have been reported as the involving signaling pathways in ICD by natural agents

There is growing interest in natural compounds and their derivatives for developing new anticancer agents. Natural compounds have a wide variety of properties, such as low toxicity, relative cheapness, and availability [59]. Therefore, discovering natural anticancer compounds with ICD-inducing capacity is a promising approach to cancer therapy. Figure 3 demonstrates how natural compounds participate in signaling cascades of ICD. Generally, ICD-inducing natural compounds are categorized into plant-, marine-, and bacteria-based groups. In the following sections, the specific properties of each compound are discussed in detail [60].

#### Plant-derived compounds

**Capsaicin**, a highly aromatic alkaloid in chili peppers, is known as one of the natural ICD inducers. It has several pharmacological functions, including improved immunity, decreased blood pressure, and reduced inflammation and pain. Capsaicin can induce ICD through surface exposure to CRT and the release of HSP90, HSP70, and ATP [61, 62]. Capsaicin has been shown to enhance ROS generation and ER stress in various cancer cell lines [63]. Capsaicin activates DCs by binding to vanilloid receptor 1 or TRPV1 [64] and affects cytokine secretion within the tumor environment, reducing immunosuppressive cells at the tumor site. Furthermore, apoptosis and autophagy can be induced by capsaicin through the reduction of phosphorylated signal transducer and activator of transcription 3 (P-STAT3), as p-STAT3 contributes to the downregulation of antiapoptosis molecules [62].

**Alternol** is a microbial fermentation product obtained from microorganisms in the bark of the yew tree. Alternol exposure has been found to cause cancer cells to develop a profound oxidative stress state, in addition to apoptosis. It has been shown that the xanthine dehydrogenase/ Xanthine oxidase (XDH/XO) can be activated in alternol-treated cancer cells, resulting in ROS accumulation and apoptosis. Alternatively, alternol-driven ICD can be inhibited by inhibiting ROS production [65]. Researchers have found that alternol induces ICD in prostate cancer cell lines by increasing DAMPs levels (HMGB1, ATP release, and CRT translocation; pro-inflammatory cytokines such as IL-1 A, IL-1B, IL-8, and IL-6) and stimulating the immune response against tumors in prostate cancer cells. Treating cancer cells with alternol activates the uptake of DCs cross-presentation and tumor-associated antigen, leading to an antitumor immune response [66].

**Curcumin** (also called curry powder) has been used for centuries as a treatment for inflammatory diseases [67]. Combination immunotherapy with curcumin is a potent inducer of ICD in tumor cells, which enhances the immunogenicity of tumors and makes cancer more susceptible to the antitumor T cell effect. Researchers have identified curcumin as an ER stressor and a strong inducer of ICD [68]. Curcumin-treated CT26 cells undergo apoptosis and release DAMPs, particularly CRT, HSP90, HMGB1, ATP, and IL-1. A combination of irinotecan and curcumin demonstrated synergistic antitumor effects on CT-26 colon carcinoma cells, accompanied by upregulation of ICD hallmarks, such as CRT and HMGB1. This combination treatment is more effective than individual treatments [69].

**Silibinin** is a flavonoid extracted from milk thistle that stimulates apoptosis and inhibits p-STAT3 in most cancerous cells [70]. Previous studies have revealed that inhibition of p-STAT3 can enhance chemotherapy-induced ICD [71]. In a recent survey, ICD was effectively induced in B16F10 and CT26 cells by silibinin and was enhanced in cells treated with a combination of silibinin and DOX. Induction of ICD is accompanied by the production of DAMPs, including HSP70, HMGB1, CRT, and the secretion of IL-12 from functionally matured DCs [43].

**Wogonin** is a flavonoid compound found in different plants, such as Scutellaria baicalensis Georgi. It possesses anti-inflammatory, antioxidant, antitumor, and immunomodulatory properties. Wogonin was demonstrated to have potent antitumor immunity in vivo [72]. Wogonin can induce ICD via CRT translocation, annexin A1, HMGB1, and ATP release [73].

**Ginsenoside** is a natural compound derived from ginseng that has been used in ancient times to treat some diseases. Rg3 is a ginsenoside that has been found to eradicate tumor cells. Additionally, it exerts modulatory effects on the immune system [74]. Son et al. reported that Rg3 could kill tumor cells via immunogenic (melanoma cell line) and non-immunogenic (lung carcinoma cell line) mechanisms by inducing apoptosis. Surface exposure to ICD markers, including CRT and HSPs, was enhanced in the Rg3-dying tumor cells. The increased expression of CRT was attributed to the uptake of dying tumor cells by DCs [75]. In addition, quercetin has been shown to significantly increase ICD efficacy induced by Rg3 by generating ROS [76].

**Resveratrol** is a natural non-flavonoid polyphenol compound found in grape leaves that possesses numerous beneficial effects, including anti-aging, anticancer, antioxidant, cardioprotective, and neuroprotective [77]. Moreover, another study showed that resveratrol exerted antitumor effects against ovarian carcinoma. Resveratrol treatment suppresses proliferation and induces apoptosis in ovarian carcinoma cells. This is mediated by cell surface exposure to CRT, secretion of HMGB1, and release of ATP. Vaccination of ID8 cells (mouse ovarian carcinoma cell line) with resveratrol significantly suppressed tumor growth in inoculated xenograft tumors. In addition, an increase in mature DCs and cytotoxic T cells was observed in xenograft tumors following resveratrol treatment, which inhibited TGF-β expression and triggered IL12 and IFN-γ secretion [78].

**Camphene** is an essential oil from the branches of Piper cernuum that induces apoptosis in melanoma cancer models. Camphene may induce ICD in apoptotic tumor cells by inducing ER stress and increasing HMGB1 and CRT expression [79].

**Alantolactone** is a natural product that is extracted from Chinese medicinal plants. This compound promoted antitumor responses through ICD induction. According to Zhang et al., alantolactone alone induces ICD in microsatellite-stable colorectal cancer. In contrast, quercetin enhances this process through ROS production and interference with the protein kinase and nuclear factor kappa B (NF-κB) pathways. The combination of alantolactone and quercetin at a molar ratio of 1:4 induced synergistic ICD. The micellar delivery of alantolactone and quercetin results in prolonged blood circulation and enhanced tumor accumulation. Indeed, combination therapy increased CRT exposure in tumor cells and the release of HMGB1. This formulation prevents the release of IL-1β, IL-10, CCL2, and TGF-β. Collectively, this formulation significantly inhibited tumor growth in a colorectal cancer model, which was mediated by ICD induction, cell toxicity, extended anti-tumor immune effects, and modulation of the immune-suppressive tumor environment [80].

**Gallotannin**-rich fractions extracted from Caesalpinia Spinosa are natural compounds that promote apoptosis through the activation of caspases 3 and 9 and externalization of annexin V. This compound has antiproliferative effects on melanoma cells. Additionally, it induces ICD markers such as ATP and HMGB1 and activates autophagy. Gallotannin can result in a reduction in the B16 melanoma tumor volume through DCs activation and an increase in CD8^+^ IFN-γ^+^ T cells [81, 82].

**Shikonin** is a phenolic compound extracted from Lithospermum erythrorhizon. Shikonin, an anti-inflammatory and antitumor phytochemical, can be used as an adjuvant for DC-based cancer vaccines via ICD induction and can enhance the expression levels of all five DAMPs in tumor cell lysates. Shikonin efficiently activated apoptotic pathways. In this context, Shikonin-treated B16F10 cells induced caspase 8 and 9, Bax, and cytochrome C apoptotic death pathways [83]. Lin et al. provided evidence that shikonin can effectively induce ICD, and this effect may serve as an adjuvant for use in DC-based cancer vaccines. In other words, shikonin can enhance the immunogenicity of vaccines via ICD [84].

**Lentinan** is a natural polysaccharide extracted from shiitake mushroom. This compound has been shown to have apoptotic effects on tumor cells. Wang et al. reported ICD induction in murine H22 Cells upon incubating with lentinan. Lentinan induced the expression of CRT, HMGB1, ATP, and HSP70. Indeed, the antitumor effect of lentinan may be correlated with the regulation of ICD-related markers, which may be beneficial for the development of liver cancer vaccines [85].

**Chalcones** are considered precursors of all flavonoids in plants that possess numerous biological activities [86]. It has been reported that JA3 and JA7, two aldehyde biphenyl chalcones, have cytotoxic effects on solid cancers and hematological malignancies. In this context, CRT exposure and the release of ATP lead to immunogenic-related responses and apoptosis. These compounds can induce ICD with severe mitochondrial damage downstream of ER and oxidative stress. They also improved the anti-leukemic efficacy of cytarabine and vincristine in different leukemic cells [87].

**Celastrol** is a promising medicinal compound with various properties such as anti-obesity, anticancer, and anti-inflammatory properties. It has been reported that celastrol at a very low dose could effectively induce ICD (without inducing toxicity) by promoting autophagy, modulating TME, and enhancing systemic immunotherapy [88, 89].

**Bullatacin** is one of the most promising antitumor agents isolated from Annona atemoya’s fruit. The compound is effective against lung, liver, breast, bladder, cervical cancer, and lymphoma. Fan et al. showed that low concentrations of bullatacin led to significant accumulation of CRT and HSP90, (as biomarkers of ICD) on the cell surface of colon cancer cell lines SW480 and HT-29 cells, indicating that bullatacin can stimulate immunogenic tumor cell death by activating ER stress [90].

**OT52** is a natural compound that belongs to coumarin compound extracted from various plants. Coumarins are natural agents with antioxidant, anticancer, and anti-inflammatory properties that regulate the inflammatory response. OT52 has shown antiproliferative effects in non-small cell lung cancer (NSCLC) cells. This cytostatic effect is attributed to ER and Golgi stress, which leads to metabolic alterations and the inhibition of STAT3 transactivation. A dose-dependent increase in CRT expression was detected with OT52. Additionally, combining BH3 protein inhibitors with OT52 resulted in a significant increase in HMGB1 release compared with OT52 alone [91].

**Hemidesmus indicus** is a widely used medicinal plant that can stimulate ICD. Turrini et al. showed that hemidesmus triggers tumor cell cytotoxicity, which is characterized by surface exposure to calreticulin and increased levels of HSP70, ATP, and HMGB1. These findings show that Hemidesmus indicus, an inducer of ICD, has the potential to be used in innovative cancer immunotherapy [92].

**Plumbagin** is a plant-derived naphthoquinone. The phenanthraquinone compound dihydrotanshinone I (derived from Salvia miltiorrhiza) enhanced ICD by producing ROS. It has been reported that nano co-delivery of these compounds significantly enhanced the half-life and tumor-targeting ability of these two drugs in orthotopic hepatocellular carcinoma (HCC) mice; consequently, this nanoformulation loaded with low doses of plumbagin and dihydrotanshinone I resulted in longer survival of HCC mice without cytotoxicity signs [93].

**Cardiac glycosides** (CGs), also known as type 1 ICD inducers, are classified into two groups, cardenolides and bufadienolides. Most CGs have natural sources and are found in plants. CGs exert several immunomodulatory functions associated with the suppression of T-helper cell activity or modulation of immune response-related genes by inhibiting NF-κB. The FDA has approved CGs such as digoxin to treat arrhythmias and heart failure. Recent studies have shown that CGs are powerful anticancer agents. Four CGs have been identified as the most effective ICD inducers: digoxin, digitoxin, ouabain, and lanatoside [94]. In 2012, Menger et al. reported that CGs could induce ICD. CGs have been shown to induce ICD biomarkers, such as CRT, HMGB1, and ATP, in several human cancer cell lines. It has also been confirmed that CGs induces ER stress. Moreover, antioxidants can inhibit the cytotoxic effects of CGs, indicating a strong correlation between their cytotoxicity and ICD induction [95]. Another report by Xiang et al. showed that co-administration of digoxin and cisplatin prodrug effectively led to a series of events in the B16F10 cell line, including ICD induction, DCs maturation, CD8 + T cell activation, and complete tumor elimination [96]. CGs show synergistic effects owing to their impact on ICD. One of the primary factors contributing to this feature is their capacity to modulate Mcl-1 and their moderate effect on Bcl-xL and Bcl-2 expression [97]. Researchers have demonstrated that digitoxin synergistically activates thapsigargin and simvastatin in estrogen-positive breast cancer cells [98]. Oleandrin treatment, a natural compound belonging to the CGs family results in secretion of ATP, HMGB1, HSP70, and HSP90 as ICD markers. As a result of oleandrin treatment, DCs were more likely to mature and activate, which further increase the efficiency of CD8^+^ T T-cell cytotoxicity. Animal models have shown that oleandrin prevents tumor growth. Oleandrin stimulated ER stress and ICD primarily through the PERK/elF2α/ATF4/CHOP pathway in the breast cancer cell lines [99].

**Vesiculated Α-Tocopheryl Succinate** is non-toxic vitamin E analog extracted from various type of seeds. Ramanathapuram et al. showed that Vα-TOS might utilize a dual approach to enhance DC-mediated cancer immunotherapy as follows: destroying tumor cells directly and maturing DC via HSPs as a danger signal [100].

**Micheliolide** is a natural guaianolide sesquiterpene lactone that induces ICD-associated DAMP molecules, such as CRT exposure, HMGB1 release, and ATP secretion. Micheliolide induced ICD by DCs maturation and activation of CD4 + and CD8 + T-cells responses in a mouse model. Indeed, the ICD-associated effects of micheliolide rely on the generation of ROS-mediated ER stress [101].

**Norcantharidin** is the most important analogs of cantharidin. Cantharidin is a natural toxin with potent anti-tumor properties. Norcantharidin was shown to induce ICD in bladder cancer cells that accompanied by promoting DC maturation and CRT exposure, but not ATP secretion [102, 103].

**Schweinfurthin** was found in Macaranga schweinfurthii (an African plant). Schweinfurthin can stimulate ICD without inducing ER stress or caspase-related mechanisms. This compound induced cell surface exposure to CRT and enhanced phagocytosis of tumor cells by DCs in vitro. Schweinfurthin does not require PERK to induce CRT exposure. It did not elicit ERp57 exposure, and a lack of ERp57 expression did not decrease CRT exposure. For this phenomenon to occur, the ER-Golgi transport system must be intact [104].

**Withania somnifera**, mostly known as Ashwagandh, belongs to the Solanaceae family. It possesses anti-inflammatory, immunomodulatory, and anticancer cancer properties [105]. It has been shown that withania has a strong potential to induce ICD in lung adenocarcinoma cancer cells [106].

**Colchicine** is a microtubule-depolymerizing drug extracted from Colchicum autumnale. This compound can induce ICD in cancer cells by affecting the expression of DAMPs, such as HSP70, HSP90, and HMGB1, without affecting the expression of CRT [107].

#### Marine-based compounds

The sea covers a large part of the Earth’s surface and is a large reservoir of biological diversity. Because of the challenging and dynamic environment in which these organisms live, they are a great reservoir of biologically active molecules that are uncommon on land. We have summarized several marine-based compounds mentioned as having ICD-inducing properties. These compounds may be a helpful approach for preventing and treating cancer, alone or in combination with other immunotherapy strategies. In addition to the various compounds produced by microalgae, some other compounds may have health benefits. There is evidence of immunomodulatory and anticancer effects of compounds derived from microalgal sources; however, ICD induction remains unknown. Various studies have been performed on human cancer cell lines, indicating that fractions and microalgae extracts can stimulate cell death through specific signaling pathways [63].

**Sulfavants** are a group of synthetic sulfoglycolipids that mimic the natural α-sulfoquinovosides found in the diatom Thalassiosira weissflodgi. At micromolar concentrations, Sulfavant A prototype of the sulfavant family, is a potent stimulator of DC maturation [108]. This agent stimulates the expression of co-stimulatory molecules and MHC II, especially CD54, CD86, and CD83, leading to T-cell differentiation. These properties demonstrate the potential of Sulfavant A as an ICD inducer. In a murine model of a melanoma vaccine, Sulfavant A has already been shown to be effective, and its efficacy has already been confirmed in preclinical studies [109].

**Alexandrium minutum** is an isolated glycopeptide from the marine dinoflagellate that has been shown to induce mitophagy in cancer cells without affecting normal cells. This form of microautophagy promotes a cascade of ICD via lysosomal ATP secretion. Researchers indicated that this compound had a potent cytotoxic effect on the A549 lung adenocarcinoma cell line, with an IC50 = 1.3 µg·mL − 1 [110].

**Docosahexaenoic acid (DHA)** is a ω-3 polyunsaturated fatty acid present in fish oil. It increases the cytotoxicity of numerous anticancer agents, especially by generating ROS and increasing cancer cells’ sensitivity. Additionally, cardio-protective effects of DHA have been revealed, and these can be very beneficial when used in combination with DOX [111, 112]. It can also induce ICD. DHA-treated human multiple myeloma cell line (OPM-2 cells) stimulates immunogenic apoptosis and autophagy and inhibits STAT3 activation in both tumor and DCs. Immunogenic apoptosis was associated with the expression of DAMP molecules (CRT, HMGB1, HSP90) and the activation of pro-apoptotic autophagy [113].

**Polyunsaturated aldehydes** isolated from three diatoms, Skeletonema costatum, Thalassiosira rotula, and Pseudonitzschia delicatissima, can stimulate necroptosis in colon and lung cell lines The receptor-interacting protein kinase 3 (RIPK3) can be activated by immune ligands, which can initiate necroptosis. This leads to increased ATP and HMGB1 levels, which are hallmarks of ICD [114].

**Thalassia testudinum** is a polyphenol extracted from marine seagrass. Pharmacological studies have proven this compound has potent anti-inflammatory and antioxidant properties. ROS-induced apoptosis of cancer cells is one of the most widely recognized mechanisms underlying the cytotoxicity of polyphenols. Prior reports state that T. Testudinum extract raises the cytosolic Ca2 + level, producing ROS and DNA fragmentation [115]. This compound inhibits colorectal cancer growth, motility, and angiogenesis by inducing ICD pathways and autophagic stress [116].

**Lepadin A** is a marine alkaloid that possesses the potential to induce ICD. At micromolar concentrations, leptin A displayed cytotoxic effects against cancer cells, which were associated with the maturation of mouse DCs. This alkaloid over-expresses MHC-II and its co-stimulatory molecules, which play a crucial role in differentiating naïve T cells by DCs, together with an increase in the effective immune response [117].

**MHO7** is a marine-derived molecule that acts as a potent ICD inducer via the ER stress-C/EBP-homologous protein (CHOP) cascade to treat triple-negative breast cancer (TNBC). MHO7 alters the expression of genes associated with ribosomes and ER proteins, resulting in ROS generation and glutathione reduction. TNBC cells are affected by MHO7 through the induction of ER stress and ROS production, release ICD-related DAMPs, and stimulate in vivo immunity by the production of antitumor cytokines, including TNF-α, IFN-γ, IL-1β, and IL-6 [118].

**Spirulina maxima and Schizochytrium sp.** microalgal compounds modulate the microbiota. Recent studies have revealed that administering microalgal species like Spirulina sp as a food supplement exhibits immunomodulation properties. Spirulina-derived modified pectin can induce mucin, IFN-α, and IL-6 releases during inflammation, triggering ICD activation [119]. The microbiota is stimulated, and mucin is released by Schizochytrium’s polyunsaturated fatty acids (PUFAs). Recent studies showed how PUFAs disrupt the organization of membrane domains by altering lipid rafts and activating particular signaling networks through stimulating microbiota, cytokine release, and anti-inflammatory activities [63].

#### Bacterial-based compounds

Several studies have demonstrated the potential of bacterial compounds to induce ICD in cancer cells.

**Lactaptin** is a proteolytic fragment of human milk kappa-casein, which is produced recombinantly in bacteria. Troitskaya et al. reported that recombinant lactaptin-induced death in cancer cells with is associated with ICD biomarkers in vitro, including external cell exposure of CRT and HSP70 and the release of ATP and HMGB1 [120].

**Septacidin** is an antibiotic produced by Streptomyces fibriatus with the potential to induce ICD.

Engineered human osteosarcoma cells and murine fibrosarcoma cells responded to septacidin by increasing CRT exposure, ATP secretion, and HGMB1 expression [121].

**Patupilone** is one of the most important therapeutic compounds isolated from the bacterium, Sorangium cellulosum. Epothilones showed strong cytotoxic effects both in vitro and in vivo. In a mouse model, patupilone has the potential to trigger ICD and translocate CRT; therefore, it can be used to vaccinate immunocompetent mice [122].

It has been shown that the gut microbiome contributes to ICD induction (Table 1). Gut microbiota can influence the ICD procedure, but how it works remains unclear [123]. Various studies have indicated that gut microbes are promising targets for improving the efficacy of cancer therapy and reducing its harmful effects [124, 125]. Based on this review, gut bacteria have a modulatory impact on immunity and are critical for the effectiveness of anticancer drugs [126–128]. Indeed, gut microbiota may influence ICD by activating T cells and DCs. Recently, the gut microbiota has been extensively studied in cancer and immunotherapy. Due to the critical role the gut microbiota plays in cancer therapy by modulating immune responses, antibiotics decrease the anticancer activity of drugs by lowering gut microbiota levels. Therefore, gut microbiota can be used as an adjuvant for tumor immunotherapy through fecal microbiota transplantation (FMT) and probiotics [129–131].

**Table 1:** Multiple gut microbiota involved in ICD induction

Gut microbiota	ICD hallmarks	Cell line	Outcomes	Ref
Peptidoglycan of L. paracasei subp. paracasei X12	HMGB1, CRT	HT-29 cells	Decreased tumor development by ICD induction	[146]
CP-1, uropathogenic Escherichia coli from prostate	HMGB1, CRT, ATP	prostate tumor tissue	Raised the anticancer activity of anti-PD-1 immunotherapy	[147]
Bacterial ghosts from E. coli Nissle 1917	HMGB1, ATP, CRT	CT26-CRC cells	OXP’s anticancer activity via ICD induction	[148]
short-chain fatty acid (SCFAs) bacterial metabolites	ANXA1, HMGB1	DU145 cells	Preventing prostate cancer progression via ICD	[149]

### Classification of natural ICD-inducing agents tested in animal and human models

The substantial numbers of natural ICD-inducing agents have been assessed preclinically for anticancer and ICD-inducing potency in in vitro and in vivo studies. Low bioavailability and water solubility is the most important obstacle limits the oral or systematic use of several types of natural agents (such as silibinin, septacidin, alternol, curcumin, lentinan, alantolactone, oleandrin, Norcantharidin, withania somnifera) as a standard treatment. At present, in order to achieve the high efficacy, the anticancer effect of low water-soluble agents is studied in in vivo animal tumor models through insitu injection in tumor site or intraperitoneally. Formulation of these compounds in nano carriers is one of the promising strategies could enhance their bioavailability and possible toxicity caused by high doses [70, 132–135]. Table 2 presents animal models and immune related antitumor effect of each compound in details.

**Table 2:** ICD-inducing natural agents studied in animal models in vitro and in *vivo*

Natural agent	Animal model	Outcome	Ref
Lentinan	NOD/SCID (severe combined immunodeficiency) mice	Exerted a direct antitumor effect on human colon cancer in vivo. Activated autophagic cell death in vivo. Activated Ca2+-induced cell death by activating IP3R in vivo Triggered ER in vivo	[150]
	mice (BALB/c-nu)	Suppressed HT-29 tumor growth in nude mice, induced apoptosis, generation of ROS Activated Caspase-3, Caspase-8, Caspasese-9, and upregulated cytosolic Cytochrome c and the ratio of Bax/Bcl-2 Prevented NF-κB activation and increased TNF-α levels in vivo	[151]
Alternol	C57BL/6 mice and severe combined immunodeficiency (SCID) mice	Delayed tumor progression, and prolonged mouse survival triggers ICD in prostate cancer cells in vivo	[65]
Curcumin	C57BL/6 mice	Enhancing Ionizing Radiation-Induced Glioma ICD	[152]
Silibinin	C57BL/6 mice	Increasing IL-12 level	[43]
Ginsenoside Rg3	BALB/c and nude mice	The targeted co-formulation of ginsenoside Rg3 and quercetin is able to induce ICD	[153]
Shikonin	Xenograph mice model that established by K562 cells	Inhibited Cdc25 phosphatases and lead to hyper-phosphorylation of CDK1	[154]
	BALB/c mice Pancreatic cancer xenograft model	Inhibited the NF-κB pathway, decreased the expression of angiogenesis VEGF, and reducing microvessel density	[155]
Lactaptin	CBA mice (RLS allograft model)	Increasing the lifespan of the tumor-bearing mice	[156]
Septacidin	Wild-type and nude (nu/nu) C57BL/6 mice	The intra-tumoral injection of septacidin significantly reduced the growth of MCA205 fibrosarcomas	[121]
Withania somnifera	MDA-MB-231 xenografts nude (nu/nu) mice	Reducing cellular proliferation and increasing apoptosis	[157]
Norcantharidin	MB49 mouse bladder cancer model	Increasing the proportions of CD4 + T and CD8 + T cells in peripheral blood reducing tumor growth in mice	[103]
Oleandrin	BALB/C mice breast cancer model	Inhibiting tumor growth and increasing tumor infiltrating lymphocytes including DCs and T cells	[158]

Most of naturally compounds with known mechanisms of action are now either just entering or about to enter clinical studies. Capsaicin, curcumin, shikonin, silibinin, ginsenoside, resveratrol, lentinan, celastrol, withania somnifera, norcantharidin, digoxin, colchicine are among the natural agents used in the clinical trials as dietary supplement.

As of yet, there is no any clinical trials evaluating the effects of natural agents on inducing of ICD in cancer patients. However, several natural agents introduced as an ICD inducer have been used in clinical trials for their safety and anti-cancer studies. Table 3 has listed the clinically tested natural agents in human with different conditions.

**Table 3:** ICD-inducing natural agents tested in different clinical trails

Intervention	Study Title	Conditions	Location	NCT number
Capsaicin	Chest Pain Perception and Capsaicin Sensitivity	Chest Pain	Cooperstown, New York, United States	NCT02346903
	Study to Evaluate the Interest of Qutenza in Patients with Head and Neck Cancer in Remission and With Sequelae Neuropathic Pain	Head and Neck Cancer	France	NCT04704453
Curcumin	Effects of Curcumin on Markers of Cardiovascular Risk in Patients With CAD	Coronary Artery Disease	Universidade Federal Fluminense, Rio de Janeiro, RJ, Brazil	NCT04458116
	Effect of Curcumin in Treatment of Squamous Cervical Intraepithelial Neoplasias (CINs)	Cervical Intraepithelial Neoplasia	Dallas, Texas, United States	NCT02554344
Shikonin	The Role of Pyruvate Kinase M2 in Growth, Invasion and Drug Resistance in Human Urothelial Carcinoma	Bladder Urothelial Carcinoma	Department of Urology, National Taiwan University Hospital Taipei, Taiwan	NCT01968928
Silibinin	Intravenous Silibinin in Combination with Peg-interferon and Ribavirin in Non-responders	Hepatitis C	Wien, Austria	NCT00684268
	Silibinin in NSCLC and breast cancer Patients with Single Brain metastasis	Brain Metastases, NSCLC Breast Cancer	Italy	NCT05689619
	Effect of Milk Thistle Derivative Silibinin(A) as a potential Anti-obesity Agent	Overweight and Obesity, Hypercholesterolemia Hypertriglyceridemia	Universidad Católica San Antonio de Murcia, Spain	NCT05069298
Ginsenoside	Ginsenoside Improve Metabolic Syndrome	Metabolic Syndrome	Seoul, Kyeonggi-do, Korea,	NCT02034136
	The Efficacy and Safety of Ginsenoside Rg3 Capsule in Prevention of Postoperative Recurrence of Hepatocellular Carcinoma	Stage I and II Hepatocellular Carcinoma	Shanghai, China	NCT01717066
Resveratrol	Anti-inflammatory and Antioxidant Effects of Resveratrol on Healthy Adults	Chronic Subclinic Inflammation, Redox Status	Turin, Italy	NCT01492114
	Resveratrol in Preventing Cancer in Healthy Participants	Unspecified Adult Solid Tumor	Michigan, United Kingdom	NCT00098969
	Effects of Resveratrol on Inflammation in Type 2 Diabetic Patients	Inflammation, Insulin Resistance, Type 2 Diabetes Mellitus	University of Turin, Italy	NCT02244879
Lentinan	The Tolerance and Efficacy of Combined Use of Didanosine (2’,3’-Dideoxyinosine; ddI) and Lentinan in HIV-Positive Patients	HIV Infections	AJI Pharma USA	NCT00002099
Celastrol	Effect of Different Ingestion Doses of Celastrol on Human Sperm Motility	Safety Issues	Baton Rouge, Louisiana, United States	NCT05413226
Withania Somnifera	Adjunctive Withania Somnifera (Ashwagandha) for Persistent Symptoms in People with Schizophrenia	Schizophrenia	Los Angeles, California, United States	NCT03437668
Norcanthari-din	Phase I Clinical Study for Evaluation of Pharmacokinetic, Safety, Tolerance of Norcantharidin Lipid Microsphere for Injection in Patients with Solid Tumor	Solid Tumor	Shenyang, Liaoning, China	NCT04673396
Digoxin	Digoxin In Treatment of Alcohol Associated Hepatitis	Acute Alcoholic Hepatitis, Alcohol-Induced Disorders	New Haven, Connecticut, United States	NCT05014087
Colchicine	Effect of Combined Use of Ivermectin and Colchicine in COVID-19 Patients	COVID-19	Cairo, Egypt	NCT05246072

All clinical trials information generated by clinicaltrials.gov

### Combination of natural compound with each other or with chemotherapeutic agents

Researchers are paying more attention to an increasing number of naturally immunogenic-enhancing substances. Combining traditional chemotherapeutic drugs with plant extracts is a common practice that increases the potency of chemotherapeutics while decreasing their tumor-specific resistance. Natural plant extracts are now widely used to treat a variety of cancers as an effective substance to enhance drug efficacy and decrease its toxicity. Combination therapy has other important advantages in the case of direct-acting natural compounds that are required in excessive and unsafe doses to inhibit cancer cell growth or induce ICD if used alone. Besides, for inducing the adequate level of ICD often a high dosage of ICD stimulus is needed which may lead to the toxicity of normal cells [136].

Curcumin is one of the robust ICD inducers and most studied natural compounds, which is mainly known for its chemopreventive effects. It has been reported that curcumin acts as an anticancer agent through multiple pathways and can enhance the anticancer effect and ICD hallmarks induced by chemotherapeutics (irinotecan and paclitaxel) and radiotherapy when used in combination therapies [137, 138]. A combination of low doses of shikonin with anthracyclines in a liposomal form has been reported to induce synergistic chemo-immunotherapy and strong ICD [134]. Ginsenoside Rg3 is the other agent studied for its influential role in increasing ICD when used in combination with PDT, agents such as doxorubicin and quercetin, and immune checkpoint inhibitors (PD-L1) [76, 139, 140]. Immune checkpoints play a crucial role in the induction of TME immunosuppression, which causes lower efficacy of immunotherapeutic agents. It has been revealed that a combination of immune checkpoint inhibitors such as anti-PDL-1, anti-CTLA-4, and anti-PD-1 can increase treatment response of patients with different cancer types [141]. Table 4 presents different combination therapies by natural agents used to enhance the ICD inducing potency of drugs.

**Table 4:** Natural agents used in combination therapies to enhance ICD

Agent	Combination	Cell line	Effect	Ref
Sunitinib	Paclitaxel	MDA-MB-231 cells	Synergistic increase of apoptosis, ICD response and improving DC maturation tumor and immunogenicity	[159]
Curcumin	Irinotecan	CT-26 colon carcinoma cells	Upregulation of ICD hallmarks such as CRT and HMGB1. Enhancing the ICD effect	[137]
	Paclitaxel	MCF-7 breast cancer cells	Improving antitumor impacts of paclitaxel by increasing ROS generation Suppression of paclitaxel resistance through the blockade of P-gp	[138]
	Radiation therapy	Normoxic or hypoxic glioma cells	Increase of radiation-mediated apoptosis, CRT exposure, ATP and HSP70 releases, and ER stress	[152]
	mEHT (Modulated electro-hyperthermia) plus resveratrol	CT-26 colon carcinoma cells	Synergistic upregulation of HSP release and immune responses, that enhances anti-tumor efficacy	[160]
Silibinin	Doxorubicin	CT26 colon cancer cells B16F10 cells 4T1 Breast cancer cells	Synergistic increase of ICD induction by Doxorubicin, and elevates the expression level of CRT, HMGB1, and HSP70. Produce higher antitumor efficacy	[143, 161]
Ginsenoside Rh2	Gemcitabine	Murine pancreatic cancer	Increasing tumor immunogenicity, Reducing the level of immunosuppressive factors	[162]
Ginsenoside Rg3	Photodynamic therapy (PDT)	Glioblastoma cells	Potentiation of the effect of chemotherapy and photoimmunotherapy	[139]
	Gefitinib	NSCLC cells (H1299 and A549)	Sensitization to the treatment with Gefitinib, Enhancing Gefitinib‑mediated tumor cytotoxicity	[163]
	Doxorubicin	4T1 cells	Increasing DOX-induced ICD	[164]
	Quercetin	CT26 and HCT116 cell line	Increasing the effects of Rg3-induced ICD by elevating ROS generation	[153]
	Paclitaxel	BGC-823 gastric cancer cells	Synergistic inhibition of growth of human gastric cancer cells	[165]
Shikonin	Mitoxantrone	B16F10 cells RM1 prostate cancer cells	Improving the effects of therapeutic and cytotoxic agents	[134]
	Doxorubicin	A549 lung cancer cells	Induction of apoptosis, damaging the mitochondrial membrane integrity, decreasing ATP levels, inhibition of glycolysis, and preventing ABC transporter expression	[166]
	Arsenic trioxide	Human HCC cell lines	Synergistic anticancer effects	[167]
Alantolactone	Quercetin	CT26-FL3 cells	Quercetin enhances ICD induction characterized by CRT exposure and HMGB1 release.	80]
Lentinan	Oxaliplatin	EC-109 Esophageal Tumor Cells	Inhibition of tumor proliferation, induction of apoptosis lentinan sensitisize cells by activation of ICD	[168]
Celastrol	Mitoxantrone	desmoplastic melanoma cells	Synergistically inducing ICD	[169]
Plumbagin	Dihydrotanshinone I	Hepatocarcinoma (HCC) cell	Formation of more ROS, inducing ICD	[170]
Digoxin	Cisplatin	B16F10 cells	Induction of ICD via CRT translocation and ATP release	[96]
Docosahexaenoic acid (DHA) marine-based compound	Paclitaxel	MCF-7 cells	Significant inhibition of tumor volume growth Sensitizing tumor cells to ptx	[171]
	Oxaliplatin	HCT116 cells	Promoting oxaliplatin-mediated autophagic cell death by enhancing ER stress	[172]
	Apatinib	MDA-MB-231 cells	Increasing inhibition on cell proliferation and migration	[173]

### Synthetic chemotherapeutic agents

Conventional chemotherapeutics are generally categorized based on their mechanism of action. Some agents can increase local immunity against tumors by inducing ICD. The co-administration of chemicals that produce ICD-associated DAMPs can improve the efficacy of conventional chemotherapeutics [18]. Indeed, chemotherapy has been employed as a conventional paradigm for cancer therapy. Some of the antitumor chemotherapeutics, such as cyclophosphamide (CPA), oxaliplatin (OXA), doxorubicin (DOX), and paclitaxel (PTX), can tackle tumors by inducing ICD in cancer cells and now are using as a standard treatment in the clinic. The ability of various synthetic chemotherapeutic agents to induce ICD has been identified in detail [142]. Cyclophosphamide is one of the most broadly used alkylating agents for cancer therapy due to its immunomodulatory functions. Several mechanisms have been attributed to cyclophosphamide-mediated immunomodulatory effects, such as triggering Th2/Th1 switch, the increasing proliferation and long-term survival of lymphocytes, and enhancement of antitumor efficacy by producing soluble mediators such as cytokines. IFN-I is induced by cyclophosphamide and mediate most of the effects attributed to this drug such as preferential expansion of memory T cells [143].

It has been shown that doxorubicin could elicit immunogenic apoptosis in a caspase-dependent manner in tumor models. Tumor cells dying in response to doxorubicin showed an effective immune response, suppression of the growth of inoculated tumors, and regression of established tumors in animal models [144].

Lau et al. reported that paclitaxel, as an antitumor drug could induce ICD in ovarian cancer cells and elicit significant antitumor responses in a TLR4-independent manner [145]. Table 5 summarizes several chemotherapeutic agents that have been described as ICD inducers.

**Table 5:** Several types of synthetic chemotherapeutic agents inducing ICD

Class of compound	Compound	Mechanism	Ref
Alkylating agents	Cyclophosphamide	↑ Type-I interferons, Stimulating T and NK cell expansion, IL-17, and Th17 cells production	[174–177]
	Oxaliplatin	↑ ER stress and CRT exposure, activate cytotoxic T lymphocytes	[58, 178]
	Melphalan	HSP90 exposure IL-1β, IL-8, and IL-6 production, DCs activation and maturation	[179]
Antimetabolites	Gemcitabine	↓ Tumor growth by ICD induction with hypoxia-inducible factor-1 (HIF-1) inhibitor PX-478 combination	[180]
	5-fluorouracil (5-FU)	HMGB1 and ATP release, ↑ CRT, recruiting DCs, production of IL-1	[18, 181, 182]
	trifluridine/tipiracil	CRT exposure, HMGB1 and ATP release, A synergistic effect in ICD was achieved by combining trifluridine/tipiracil with OXP.	[183]
Anthracyclines	doxorubicin	Induced apoptosis in a caspase-dependent manner in many cancers cell line	[144]
	mitoxantrone	Promoting CRT exposure in colorectal cancer cells, induces autophagy by releasing HMGB1 and ATP in pancreatic and breast cancer cells	[184]
	Daunorubicin	Induced CRT surface expression and release of HSP70/HSP90 and IFN in AML treatment.	[63]
	idarubicin (IDA)	HMGB1, HSP70/90, and CRT exposure were detected in response to IDA treatment in several cancer cell lines.	[13]
	Bleomycin	Translocation of CRT or ERp57. HMGB1 and ATP were released from dying cancer cells as a result of bleomycin-induced autophagy	[185]
Microtubular inhibitors	paclitaxel	CRT expression in ovarian tumors	[186]
	docetaxel (on its own does not appear to be a particularly effective ICD inducer)	NSCLC cell lines exhibited the greatest levels of ATP, CRT, and HMGB1 when was combined with carboplatin or cisplatin. ↑ CTLs and ↓ Tregs in patients with breast cancer and NSCLC treated with Co-administration of vinorelbine and cisplatin with docetaxel	[187]
Other chemo-therapeutics	bortezomib	Appearance of HSP90 on tumor cells	[188]
	crizotinib	High-dose could induce ICD in cancer cells	[189]
Other drugs	colchicine	HSP70, HSP90, and HMGB1, without affecting the expression of CRT	[107]
	Cetuximab (combined with FOLFIRI)	Letting DCs phagocytose tumor-dying cells and triggering an immune response by CD8 + T cells. Panitumumab or cetuximab alone induces ICD in DiFi cells	[190]

#### Metal-based agents inducing ICD

There has been a significant increase in the discovery of small molecules as potential inducers of ICD. Metal-based agents have been shown to induce ICD. Anticancer metal compounds have been shown to elicit an immune-related response to ICD induction in tumor cells. There is a substantial increase in the number of molecules being tested as potential ICD inducers such as metal-based complexes. Metal-based drugs are based on Ru, Pt, Ir, Cu, and Au, which have the potential to induce ICD and elicit an immune response against tumor cells. The success of platinum containing molecules (e.g., cisplatin, carboplatin and oxaliplatin) as an anticancer agent leads to the investigation in this field. Several non-platinum metal-based agents such as ruthenium-based compounds can be used as an alternative to platinum-anticancer agents [58]. With this notion, the employment of novel anticancer metal agents with the potential to trigger ICD shows promise as new immunotherapies for cancer as an emerging approach in the treatment of neoplasia. Table 6 shows the metals and their mechanisms of action in tumor cell death mediated by ICD.

**Table 6:** Several types of metal-based anticancer agents as inducer of ICD

Compound	Study phase	NCT number	Mechanism	Ref
Oxaliplatin	I/II	NCT04068610	CT26 were treated with OXP were exposed to pre-apoptotic CRT. The HMGB1 release was observed in cells treated with OXP. Findings indicate that OXP can promote immunogenic lung cancer cell death on LLC cells. OXP induces ICD in glioma cells. OXP exposure led to eIF-2a phosphorylation.	[191, 192]
Ruthenium complexes	NG^*^	NCT04577742	KP1339 could trigger ICD markers in vitro like CRT, ATP, and HMGB1 In colon cancer cell lines, plecstatin-1 was also a Ru (II) arene complex with ICD induction properties by ↑ HSP70/90	[193, 194]
copper (I/II) complexes	I	NCT02963051	↑ ROS levels resulting in ER stress	[195]
Iridium (III) complexes	III	NCT00548600	Bis(2-chloroethyl)-azane moiety in an Ir (III) complex could induce ICD in non-small cell lung cancer	[196]
gold (I) complexes	NG^*^	NCT04907422	The compound Arambula et al. reported is redox-active and induces ICD	[197]

All clinical trials information generated by clinicaltrials.gov. *NG:Not given

## Conclusion

Based on the known mechanisms of ICD induction and the related molecular mechanisms, ICD induction is a promising area of research. Natural compounds that act as ICD inducers could represent a new frontier in cancer therapy. Among them, plant-derived, marine molecules and bacteria-based compounds may represent attractive modalities for ICD induction.

Indeed, only a small number of anticancer chemicals or natural compounds have been found to induce ICD. The identification of new compounds that induce ICD will be important in the future. Most of the known phytochemical compounds are able to induce apoptosis, ER stress, and ROS production in cancer cells. We believe that these phytochemicals are promising agents in immunotherapy of cancers due to the broad spectrum of anticancer effects and are potential candidates to be studied for their ICD capability. Thus, there is an urgent need to explore the potential of novel natural compounds to induce ICD, whether alone or in combination. A combination of natural ICD inducers with immune checkpoint inhibitors or other chemotherapeutic drugs could be one of the combinational therapy strategies for tumor regression through overcoming TME immunosuppression and enhancing tumor immunogenicity. Moreover, several types of plant- and/or marine-based compounds, indicated outward properties of ICD induction in vitro, need to be observed for their antitumor immune response in vivo. Herbal formulations mainly consist of several bioactive molecules, which have been shown to be beneficial for various types of diseases. However, they have not been fully accepted as standard treatment, mainly due to their complexity and lack of strict quality control during preparation. Moreover, most of the naturally derived compounds have low water solubility and bioavailability that limit their clinical usage. In this regard, nano-formulation of low water-soluble agents could be the promising approach and not only enhances the agent‘s water solubility and bioavailability but also could increase the ICD potency of natural agents. In the case of drug combinations, molecular interactions in complex phytochemicals can be antagonistic or dangerous because herbal formulations may contain toxic or even lethal compounds. In addition, only a few natural cytotoxic drugs capable of inducing ICD are being investigated in clinical trials. Therefore, the identification of other chemicals and phytochemicals that may cause ICD is clinically important. It seems that more researches are desired to determine the guidelines for the clinical application of natural ICD inducers.

## Data Availability

The datasets used and/or analyzed during the current study are available from the corresponding author on reasonable request.

## Abbreviations

ICD: Immunogenic cell death
DAMPs: Damage-associated molecular patterns
DCs: Dendritic cells
NKs: Natural killer cells
APCs: Antigen-presenting cells
CTL: Cytotoxic T lymphocytes
PAMPs: Pathogen-associated molecular patterns
PRRs: Pattern recognition receptors
CRT: Calreticulin
HMGB-1: High mobility group box-1
ATP: Adenosine triphosphate
HSPs: Heat shock proteins
TME: Tumor microenvironment
WHO: World Health Organization
TNF-α: Tumor necrosis factor-α
TLR: Toll-like receptor
ERp57: ER-resident protein 57
Hyp-PDT: Hypericin-photodynamic therapy
PERK: Protein kinase-like ER kinase
LAMP 1: Lysosomal-associated membrane protein 1
PANX1: Pannexin 1
NLRP3: NLR domain-containing protein 3
CXCL10: Chemokine ligand 10
FPR1: Formyl peptide receptor 1
ANXA1: Annexin A1
XDH/XO: The xanthine dehydrogenase/ Xanthine oxidase
P-STAT3: Phosphorylated signal transducer and activator of transcription 3
NSCLC: Non-small cell lung cancer
HCC: Hepatocellular carcinoma
CGs: Cardiac glycosides
RIPK3: Receptor-interacting protein kinase 3
TNBC: Triple-negative breast cancer
